# AAV Vectors Pseudotyped with Capsids from Porcine and Bovine Species Mediate In Vitro and In Vivo Gene Delivery

**DOI:** 10.3390/v16010057

**Published:** 2023-12-29

**Authors:** Darrick L. Yu, Laura P. van Lieshout, Brenna A. Y. Stevens, Kelsie J. (Jagt) Near, Jenny K. Stodola, Kevin J. Stinson, Durda Slavic, Sarah K. Wootton

**Affiliations:** 1 Department of Pathobiology, University of Guelph, Guelph, ON N1G 2W1, Canada; 2 Animal Health Laboratory, Laboratory Services Division, University of Guelph, Guelph, ON N1G 2W1, Canada

**Keywords:** adeno-associated virus (AAV), novel serotype, porcine capsid, bovine capsid, luciferase imaging, transduction

## Abstract

Adeno-associated virus (AAV) vectors are among the most widely used delivery vehicles for in vivo gene therapy as they mediate robust and sustained transgene expression with limited toxicity. However, a significant impediment to the broad clinical success of AAV-based therapies is the widespread presence of pre-existing humoral immunity to AAVs in the human population. This immunity arises from the circulation of non-pathogenic endemic human AAV serotypes. One possible solution is to use non-human AAV capsids to pseudotype transgene-containing AAV vector genomes of interest. Due to the low probability of human exposure to animal AAVs, pre-existing immunity to animal-derived AAV capsids should be low. Here, we characterize two novel AAV capsid sequences: one derived from porcine colon tissue and the other from a caprine adenovirus stock. Both AAV capsids proved to be effective transducers of HeLa and HEK293T cells in vitro. In vivo, both capsids were able to transduce the murine nose, lung, and liver after either intranasal or intraperitoneal administration. In addition, we demonstrate that the porcine AAV capsid likely arose from multiple recombination events involving human- and animal-derived AAV sequences. We hypothesize that recurrent recombination events with similar and distantly related AAV sequences represent an effective mechanism for enhancing the fitness of wildtype AAV populations.

## 1. Introduction

AAV vectors are widely regarded as ideal candidates for in vivo gene therapy because of their biology; they readily transduce a wide range of dividing and non-dividing cells, they are replication-defective and their genomes reside episomally in transduced cells, they are minimally immunogenic, they elicit sustained transgene expression, and they have a strong safety profile [1]. However, because of the high prevalence of adenovirus infections in the human population, there is a correspondingly high level of seropositivity against AAVs of different serotypes [2]. This high rate of pre-existing immunity can limit the usefulness of many human AAV serotypes, as this can potentially compromise transgene expression by blocking transduction [3,4] and, in many cases, is an exclusion criterion for clinical trials, particularly those in which the vector is to be delivered systemically [5,6]. To circumvent this, recombinant AAV (rAAV) vectors may be altered by pseudotyping. In this process, a vector can be packaged into the capsid of a heterologous AAV. For example, a transgene flanked by AAV2 ITRs can be packaged into a capsid from bovine AAV [7,8]. The result is an rAAV particle with a relatively low to nonexistent seroprevalence in a target population, with the potential for vastly improved transduction efficiency. Since the capsid determines tissue tropism, it is possible to alter the range of tissues targeted simply by changing the vector’s capsid. 

Significant advancements have been made in the field of AAV capsid engineering using rational design, directed evolution, combinatorial libraries, and in silico reconstruction of ancestral capsid variants [9]. These approaches have led to the development of capsids that evade recognition by the anti-AAV antibodies typically found in the human population and have enhanced transduction of target tissues of human origin. Another approach to evading pre-existing immunity is to isolate AAVs from non-human species [10]. For instance, AAVrh10 and AAVrh74 are recombinant vectors originating from rhesus macaques. A survey of healthy adults revealed that 59% of individuals had detectable antibodies against AAVrh10, and 21% had neutralizing antibodies [11]. Analysis of antibodies against AAVrh74 in patients with Duchenne muscular dystrophy and limb girdle muscular dystrophy revealed that 83% were seronegative [12]. However, the isolation of novel AAVs from more distant species could yield AAVs that are not neutralized by human sera. Animal AAVs have been detected in canine, bovine, ovine, caprine, porcine, and avian species, although when considering the prevalence of adenovirus in animal species, there remains a wealth of as yet undiscovered AAV isolates [13,14,15,16,17]. As such, the purpose of this research was to identify and isolate novel AAVs from animal tissues for use in the development of new gene therapy vectors with low seropositivity in the human population.

## 2. Materials and Methods

Sources of samples. Fresh lung, liver, ileum, and distal colon samples from a range of mammals were collected through a partnership with the University of Guelph Animal Health Laboratory (AHL) post-mortem (PM) and bacteriology departments. Animals were necropsied on site and sampled at the time of necropsy in the PM room. Samples which were necropsied off site were sampled upon arrival to the bacteriology lab; however, only exotic species not regularly seen in the PM room were taken from this source. Genomic DNA from the colon of 164 healthy pigs, part of a study on sub-clinical infections and biomarkers in Ontario swine, was also provided by the AHL bacteriology department. 

DNA extraction—QIAGEN kit. Except for the porcine colon samples, DNA extractions from all samples were performed using the QIAGEN DNeasy Blood and Tissue extraction kit, following the animal tissue spin-column protocol described by the manufacturer. Briefly, tissues were lysed by adding 25 mg of tissue to 180 µL of buffer ATL, as per the QIAGEN kit protocol, with an overnight incubation at 56 °C after addition of 20 µL proteinase K. After extraction, DNA was quantified using a NanoDrop 2000 (Thermo Scientific, Mississauga, ON, Canada) and found to be, on average, 100 ng/µL.

DNA extraction—MagNA Pure robotic extraction. Porcine colon samples were extracted using the MagNA Pure extraction robot (Roche, Mississauga, ON, Canada). Tissues were lysed by adding 100 mg of tissue to 900 µL of Tripure with 2 stainless steel grinding balls (Montreal Biotech, Dorval, QC, Canada) and homogenized for 3 min at 30 hz using a MM 300 mixer mill (Retsch, Newtown, PA, USA). Sample tubes were briefly centrifuged to sediment large pieces of tissue, and 200 µL of supernatant was loaded into the MagNA pure. The extraction was performed using the Roche DNA Isolation Kit I and the High-Performance Blood Cells protocol on the MagNA Pure, with a 200 µL sample volume and a 100 µL elution volume. After extraction, DNA was quantified using a NanoDrop 2000 (Thermo Scientific) and found to be, on average, 200 ng/µL.

**Figure 1:**
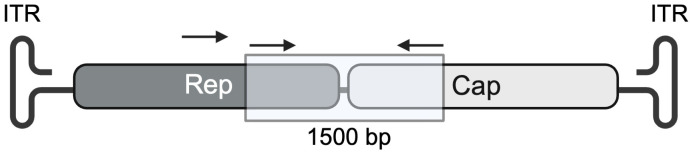
Location of PCR primers used for detecting AAV sequences. Primers used for screening genomic DNA samples were designed to amplify a 1500 base-pair amplicon, which consisted of the 3′ end of the rep gene and the 5′ end of the cap gene. Hemi-nested PCR using degenerate primers for a highly conserved region of the genome allowed for the detection of low quantities of AAV DNA.

Screening of genomic DNA for presence of AAV genomes by polymerase chain reaction (PCR). A hemi-nested PCR reaction using degenerate primers was used to screen for AAV genomes in the genomic DNA samples. This method was an adaptation of that employed by Katano et al. (2004), implementing the use of a nested forward primer in a second PCR reaction and allowing for the detection of the low concentrations of AAV DNA integrated with a host genome [18]. Table 1 shows the PCR conditions and primers used for both reactions. PCR Platinum Supermix (Invitrogen, Burlington, ON, Canada) was used as the reaction master mix. The plasmid pDGM6 [8] containing the rep region of AAV2 and the cap region of AAV6 were used as a positive control, and RNAse free water was used as a negative control. Analysis of PCR products was performed by gel electrophoresis on a 0.8% agarose gel stained with ethidium bromide. The desired amplicon, containing the 3′ end of the rep region and the 5′ end of the cap region, was 1500 bp (Figure 1). PCR products with a band between 1400 and 1600 bp were selected as potential positive samples. For samples with high levels of non-specific binding, the target band was excised and recovered using the Direct-gel-spin DNA recovery kit (Applied Biological Materials).

pGEM-T easy vector cloning of PCR products. PCR products of the correct size were directly ligated into the pGEM-T easy vector (Promega), as per the manufacturer’s instructions, using 3.5 µL of PCR product with incubation at 4 °C overnight. After incubation, 4 µL of ligation reaction mixture was transformed into Escherichia coli GT116 competent cells (InvivoGen), and plasmid DNA was extracted from cultures of isolated colonies using the Plasmid DNA Miniprep Kit from Bio Basic.

DNA sequencing. Successful ligation was confirmed by digestion of the plasmid extraction with EcoRI and analysis by gel electrophoresis. If bands were observed at 3000 bp and 1500 bp, representing the vector and insert, respectively, the plasmid extraction was sent to the University of Guelph Advanced Analysis Center for Sanger sequencing using the M13 forward and reverse primers (located ~60 bp upstream and downstream from the vector insertion site).

Sequence analysis. Forward and reverse sequences were aligned in SeqMan Pro (DNAStar) and the plasmid sequence was removed at the EcoRI cut sites flanking the insertion. The generated consensus sequences for the entire amplicon and the rep and cap regions individually were analyzed against the NCBI nucleotide database using the BLAST Megablast algorithm. Analysis of recovered AAV isolates was performed using the integrated bioinformatics software Geneious version R11 (Biomatters, Boston, MA, USA), which was used for sequence manipulation, alignment with known sequences (ClustalW algorithm), and development of phylogenetic trees (UPGMA algorithm). Geneious was also used to generate and align protein sequences in order to analyze novel AAVs at the amino acid level. The Dual Brothers recombination plugin for Geneious was used to analyze the AAV.Po.Guelph capsid sequence for recombination events [19,20].

Cloning of the full-length porcine and bovine AAV capsids. The forward and degenerate reverse primers and PCR conditions used to amplify the full-length porcine and bovine capsid genes are shown in Table 2. Note that DNA samples were stabilized by the addition of trehalose to a final concentration of 200 mM. PCR products were pGEM-Teasy cloned and sequenced using universal T7 forward and reverse primers.

AAV vector production and purification. HEK 293 cells were transfected utilizing a PEI-based transfection protocol as previously described [21]. A four-plasmid system, including plasmids encoding rep and helper functions, pMTRep6 [22] and pLadeno5 [23], respectively, was used to generate AAV particles encoding the reporter gene human placental alkaline phosphatase (hPLAP) driven by the CAG promoter. This AAV vector genome was encoded within plasmid pACAGAP [24]. A fourth plasmid encoding AAV.Po.Guelph or AAV.Bov.Guelph capsids (pCAGαPo.GuelphCap, or pCAGαBov.GuelphCap) expressed from a truncated CAG promoter [25] provided the capability of packing the AAV vector genome into AAV.Po.Guelph or AAV.Bov.Guelph capsids, respectively. pCAGαPo.GuelphCap and pCAGαBov.GuelphCap plasmids were constructed by digesting plasmid pCAGαeGFP [25], as well as PCR products encoding AAV.Po.Guelph and AAV.Bov.Guelph, with EcoRI and BglII (NEB, Whitby, ON, Canada). An optimized Kozak sequence was inserted in front of the AAV.Po.Guelph and AAV.Bov.Guelph capsid open reading frames in order to promote translation. The porcine and bovine capsid genes were cloned into the EcoRI and BglII sites downstream of the CAGα promoter using the Capsid-EcoRI forward 5′-GC**GAATTC**GCCACC*ATGTCGTTTGTTGATCACCC*-3′ (where the bold text represents the EcoRI restriction site and underlined text indicates the Kozak sequence) and Capsid-BglII reverse 5′-GC**AGATCT**TTACAGATTGCGGGTAAGG-3′ (where the bold text indicates the BglII restriction site and the underlined text denotes the stop codon) primers and the pGEM-Teasy plasmid containing the full-length capsid gene as template DNA using standard molecular cloning techniques.

To determine virus distribution in the cells and supernatant, 1.2 × 10^6^ HEK293 cells were plated in 6-well dishes and allowed to adhere overnight. Cells were transfected with 2 μg of AAV genome (pCASI-Luciferase), 2 μg of adenovirus helper plasmid (pAdΔF6), and 2 μg of AAV helper plasmid (pAAV.Po.Guelph or pAAV.Bov.Guelph) using PEI (Polysciences Inc., Warrington, PA, USA). Supernatant and cell lysate were harvested separately on days 3 and 6 post-transfection, and vector DNA was extracted and quantified by Taqman PCR as previously described [26] to determine the relative distribution of virions between the two fractions. No significant differences were observed between vector distribution in the supernatant or lysate on day 3 or 6 post-transfection for either AAV.Po.Guelph or AAV.Bov.Guelph (Figure S1).

For iodixanol gradient purified vector preparations, cells and supernatant were pooled and freeze–thawed three times at −80 °C to lyse cells and release virus particles. In between freeze–thaw cycles, the cell lysate and supernatant were vortex mixed to facilitate separation of the virus from cell debris. After completion of the freeze–thaws, lysate was spun at 3000× *g* to pellet cellular debris, and supernatant was filtered through a Millipore Stericup 0.22 µm filter (EMD Millipore, Etobicoke, ON, Canada). Benzonase nuclease (≥500 units) (Sigma, St. Louis, MO, USA) was added to digest nucleic acids and help reduce viscosity by treatment at 37 °C for 30 min. A 100 k Omega Centramate T-SERIES 0.019 m^2^ cassette (Pall Canada Direct Ltd., Mississauga, ON, Canada) was used in an LV centramate holder to concentrate the supernatant from over 500 mL to approximately 25 mL, according to the manufacturer’s instructions. A 100 k Amicon Ultra-15 Centrifugal Filter Unit (EMD Millipore) was used to further concentrate the sample to a volume of approximately 5 mL. The concentrated sample was loaded onto an iodixanol gradient as described previously [27] and ultracentrifuged at 41,000 rpm for 3 h at 4 °C in an SW41 rotor. The fraction containing the vector was harvested by puncturing the tube slightly below (3–5 mm) the 60–40% interface with an 18-gauge needle (bevel up) attached to a 1 mL syringe [27]. A 100 k Amicon filter unit was used to exchange the iodixanol gradient solution for HBSS.

Cell culture. HEK293 (ATCC**^®^** CRL-3216™), HEK293T (ATCC**^®^** CRL-3216™), HeLa (ATCC**^®^** CCL-2™), HT1080 (ATCC**^®^** CCL-121™), PK-15 (ATCC**^®^** CCL-33™), BHK-21 (ATCC**^®^** CCL-10™), Vero (ATCC**^®^** CCL-81™), A549 (ATCC**^®^** CRM-CCL-185™), MDBK (ATCC**^®^** CCL-22™), MDCK (ATCC**^®^** CCL-34™), NRLE, and Dulac cells were maintained in Dulbecco’s modified Eagle’s medium (DMEM) supplemented with 10% FBS and 2 mm L-glutamine. AML12 (ATCC**^®^** CRL-2254™) cells were supplemented with 10% fetal bovine serum, 10 µg/mL insulin, 5.5 µg/mL transferrin, 5 ng/mL selenium, and 40 ng/mL dexamethasone (insulin–transferrin–selenium (ITS-G); ThermoFisher, Mississauga, ON, Canada). Primary culture bovine tracheal epithelial cells were established as previously described [28]. Briefly, tracheas were obtained from market-weight cattle immediately following slaughter. The tracheal mucosa was removed and incubated overnight at 4 °C in phosphate-buffered saline wash solution containing 0.1% protease (Dispase, Invitrogen, San Diego, CA, USA), 0.1 mg/mL penicillin–streptomycin, 0.5 mg/mL gentamicin, and 10 µg/mL amphotericin B. The following day, cells were harvested by scraping the mucosal surface with a scalpel blade to release the epithelial cells. Cell viability was assessed with trypan blue, and 3 × 10^5^ viable cells were suspended in 1 mL of culture medium consisting of DMEM and Ham’s F-12 medium (DMEM/F12), 5% sterile fetal bovine serum, 0.1 mg/mL penicillin–streptomycin, 5 μg/mL amphotericin B, 0.5 mg/mL gentamicin, 25 μg/mL bovine pituitary extract, 10 ng/mL epidermal growth factor, and 1% insulin–transferrin–selenium (Sigma).

In Vitro transduction assay. Cells were plated in 12-well dishes at a density of 1 × 10^5^ per well, and the next day, AAV vectors were added at an MOI of 20,000. After 72 h, cell lysates were harvested using Passive Lysis Buffer (Promega, Madison, WI, USA), and luciferase expression was quantified by luciferase assay (Promega) and normalized by protein concentration determined by Bradford assay (Fisher Scientific, Mississauga, ON, Canada).

Animal ethics statement. All experiments involving mice were approved by the University of Guelph Animal Care Committee (AUP #3827) and conducted in accordance with the Canadian Council on Animal Care (CCAC). Balb/c mice (Strain code 028) were purchased from Charles River Laboratories, and after a one-week acclimation period, AAV vectors were administered to mice at 6 weeks of age. 

AAV administration in vivo. AAV vectors were produced by quadruple transfection as outlined above and purified by iodixanol gradient centrifugation and further concentrated using Amicon Ultra-15 centrifugal filter units. Six-week-old female Balb/c mice were purchased from Charles River. Intranasal (IN) delivery was performed as previously described [29]. Intraperitoneal (IP) injections were completed using a 27-gauge tuberculin syringe, and vector was diluted in PBS to 100 μL. IV administrations were performed by tail-vein injection using a 29-gauge needle with vector diluted in PBS to 100 μL.

In Vivo luciferase imaging. Six-week-old Balb/c mice were administered 1 × 10^11^ vg of AAV.Po.Guelph-Luciferase or AAV.Bov.Guelph-Luciferase by either IN or IP routes. Mice were imaged using the IVIS Spectrum CT instrument 20 min following injection with D-luciferin given IN or IP. Living Image version 4.7.3. software was used to quantify luciferase expression.

Genbank accession numbers. The nucleotide sequences for AAV.Bov.Guelph (AAV.Bov.G-1) and AAV.Po.Guelph (AAV.Po.G-1) capsid genes were submitted to Genbank with accession numbers OR759014 (AAV.Bov.G-1) and OR759015 (AAV.Po.G-1), respectively.

Statistics. All statistical analysis was completed by two-way ANOVA using GraphPad Prism 7 software. All error bars represent standard deviation of the mean.

## 3. Results

### 3.1. Analysis of Porcine AAV Screening Amplicon

A total of 104 samples were screened for the presence of AAVs by PCR, the majority which were porcine colon samples from animals that showed no outward signs of disease. All other samples were from diseased animals submitted for necropsy. A summary of these samples is found in Table 3. The 104 PCR screenings showed 51 positive samples and included one canine, one hippopotamus, one equine, and the remainder porcine AAVs. Analysis of a rep gene fragment derived from a screening product amplified from a porcine colon sample showed 92% homology with the previously characterized porcine isolate AAV.Po1 [30]. Furthermore, AAV.Po.Guelph contained a 15-base intergenic spacer between the rep and cap fragments, shared by the AAV.Po1 clade, whereas the AAV2 clade contains the 16-base intergenic spacer more commonly observed with human-derived AAVs. 

**Figure 2:**
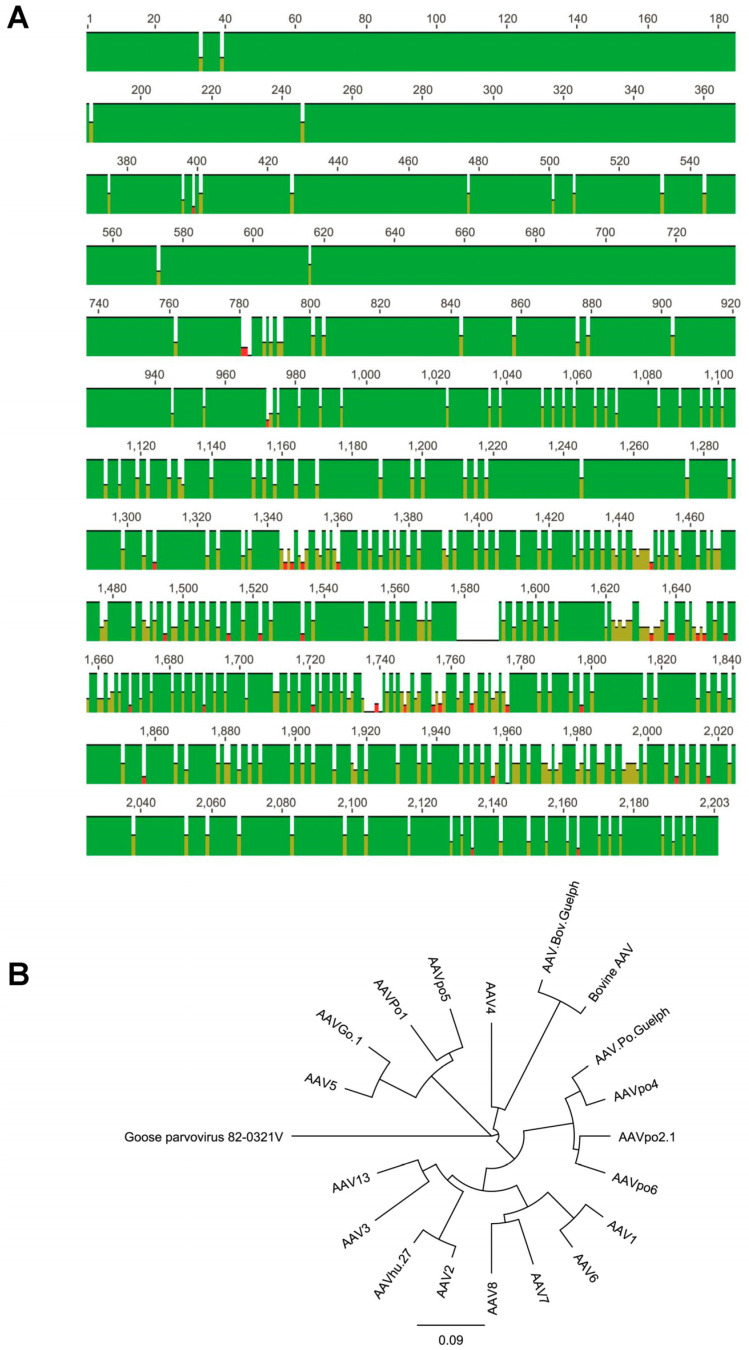
Alignment between the capsid sequences AAV.Po.Guelph, AAVpo6, AAVpo2.1, and AAVpo4. (**A**) Green indicates 100% nucleotide identity between these four sequences, while yellow indicates between 75% and 50% nucleotide identity. Red indicates 25% nucleotide identity. High homology can be observed, particularly within the first 413 nucleotides. Sequences become far more divergent towards the end of the cap gene. (**B**) Phylogenic tree showing the relative similarity of various human and animal AAV capsid nucleotide sequences. Goose parvovirus is thought to be related to adeno-associated virus and thus was used as the outgroup. Overall, AAV.Po.Guelph is most similar to AAVpo4, and both share homology with AAVpo2.1 and AAVpo6. This group shares greater similarity with human AAV serotypes than it does with the first porcine AAV capsid sequenced, AAVPo1.

A high degree of similarity exists between the AAV.Po.Guelph isolate and previously characterized porcine isolates for the first 413 base pairs (bp) of the capsid gene (Figure 2). Alignment of DNA sequences containing this region from AAV.Po.Guelph, AAVpo2.1, AAVpo4, and AAVpo6 demonstrated a high degree of conservation [31]. Overall, across the entire capsid gene, AAVPo1 and AAV.Po.Guelph share only 65.1% nucleotide identity; however, within this 413 bp segment, sequence identity was unusually high at 90.4%. This was also true of the previously discussed AAVpo2.1, AAVpo4, and AAVpo6. These isolates had a high degree of shared sequence identity with Po1 within the 413 bp region (90.4%) but, overall, only shared 65% to 66% sequence identity with Po1. In addition, AAVpo2.1, AAVpo4, and AAVpo6 demonstrated relatively high capsid gene shared sequence identity with the AAV.Po.Guelph capsid, with 88.6%, 93.6%, and 87.7% sequence identity, respectively (Figure 2A). On the other hand, another porcine isolate—AAVpo5—demonstrates a much greater level of homology with AAVPo1, at 89% sequence identity, than it has with any of the other capsids sequenced so far. A phylogenetic analysis using goose parvovirus as the outgroup demonstrated a high degree of sequence similarity between AAV.Po.Guelph and the human AAV2 capsid (Figure 2B). Interestingly, AAVPo1 and AAVpo5 cluster more closely with AAV5 and AAVGo.1, suggesting a possible shared lineage.

When comparing AAV.Po.Guelph to the next most similar capsid sequence, AAVpo4, a large number of amino acid changes were still observed [31,32]. A summary of the changes is listed in Table 4.

### 3.2. Numerous Recombination Events Have Occurred between Human AAVs and Animal-Derived AAVs to Form AAV.Po.Guelph

**Figure 3:**
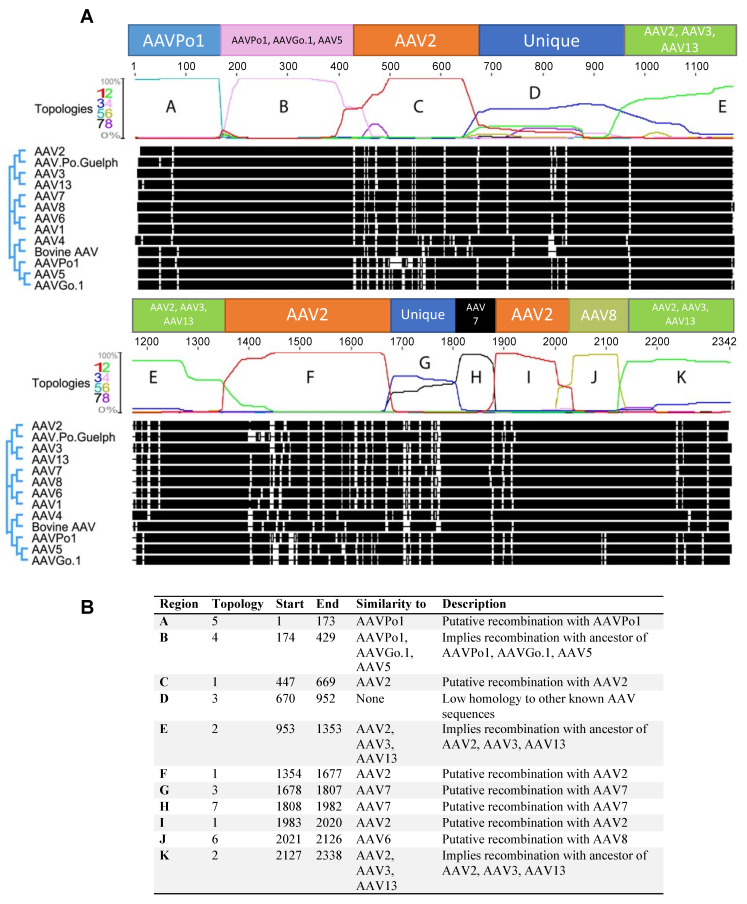
Probability of various tree topologies for AAV.Po.Guelph as predicted by the Geneious Dual Brothers recombination plugin. (**A**) The rectangular overlay above the tree topology probability graph indicates the likely origin of the different sequence regions of the capsid. For specific color-coded tree topologies (1 through 8), see Figures S2 and S3. (**B**) Description and location of topology regions A through K indicating the likely lineage of specific capsid regions.

Utilization of the Dual Brothers recombination plugin for Geneious facilitated the detection of numerous putative recombination events. Several different tree topologies were generated for different parts of the capsid gene (Figures S2 and S3). An analysis of the full-length AAV.Po.Guelph capsid was performed to identify the probability of the different topologies, showing that different topologies were favored in different regions of the capsid sequence (Figure 3A). Analysis revealed that AAV.Po.Guelph has undergone multiple recombination events with other distantly related AAV capsids, including those of different human serotypes. In fact, a large proportion of the AAV.Po.Guelph sequence appears to be derived from AAV2 or a related ancestor. A putative recombination may have occurred at position 173, as the capsid sequence preceding this point shares high homology with AAVPo1 (Figure 3A,B, region A, tree topology 5 (Figure S3).

Region B appears to have greater homology with AAVPo1, AAVGo.1, and AAV5, as tree topology 4 (pink, Figure S2) suggests recombination with a possible ancestor of these viral capsids. Region C shows high homology with AAV2, with the tree topology probability approaching 100% for this area (tree topology 1, red, Figure S2). Within region D (tree topology 3, blue, Figure S2), there did not appear to be significant homology with other known distantly related AAV capsids to imply a possible recombination event, suggesting that this is one region that drifted over time to better fit the host species. Region E (tree topology 2, green, Figure S2) shows the most homology with AAV2, AAV13, and AAV3, suggesting a possible recombination with an ancestral sequence to these serotypes. A high probability exists for a putative recombination event that occurred between an ancestral sequence to AAV.Po.Guelph and AAV2, as with region C (both in red, tree topology 1, Figure S1). Alternatively, since so much of AAV.Po.Guelph has high sequence similarity with AAV2 or related serotypes, AAV.Po.Guelph may have its origins in AAV2 or a related sequence. Next, with region G, AAV.Po.Guelph once again appears to diverge from known AAV sequences, although it appears that there may be some homology with AAV7 and AAV8, suggesting a possible recombination with AAV7 had occurred and subsequent genetic drift resulted in divergence from known capsid sequences. Once again, with region I, it appears that high sequence similarity with AAV2 is shared, as was the case with regions C and F, suggesting yet another possible recombination event or common evolutionary origin. Penultimately, region J shares much similarity with AAV8 (tree topology 6, yellow, Figure S3), suggesting that another possible recombination event gave rise to this part of the AAV.Po.Guelph capsid gene. Lastly, region K, like region E, shares similarity with AAV2, AAV3, and AAV13, suggesting a possible recombination event that had occurred in the past with an ancestor of these sequences.

### 3.3. AAV.Bov.Guelph Capsid Protein Is Highly Similar to the Bovine AAV Capsid

We obtained a caprine adenovirus isolate from the Animal Health lab, and using our screening primers, we found it to be positive for AAV. The amplified PCR product was highly similar to a previously characterized bovine AAV capsid sequence (Genbank: AY388617.1), with only two amino acid changes distinguishing it from bovine AAV: I124L and R499G. Although isolated from a caprine adenovirus stock, this capsid, named AAV.Bov.Guelph, was found to be unrelated to the previously characterized caprine AAV, AAVGo.1 (GenBank: AY724675.1). 

### 3.4. AAV.Bov.Guelph Transduces Mammalian Cell Lines More Efficiently Than AAV.Po.Guelph

**Figure 4:**
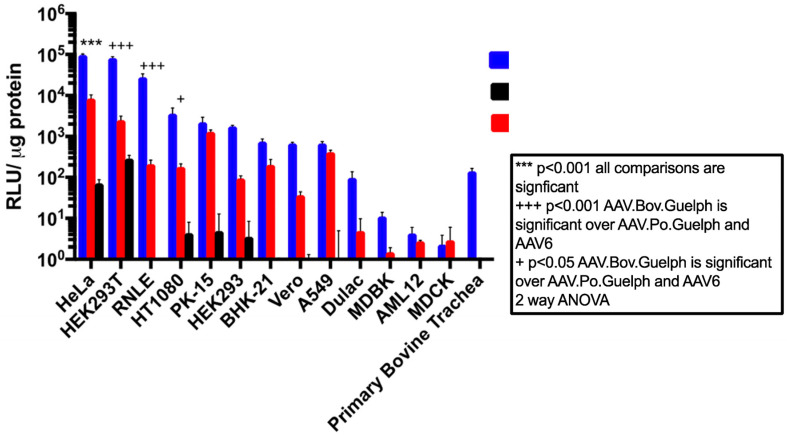
In vitro transduction profile of AAV.Po.Guelph, AAV.Bov.Guelph, and AAV6 in a panel of mammalian cell lines. A total of 50,000 cells in a 24-well plate were transduced with AAV at an MOI of 20,000, and 72 h post-transduction, cell lysate was harvested using passive lysis buffer and luciferase expression was quantified via luciferase assay. Bradford assays were used to normalize protein concentration. A two-way ANOVA was used to determine statistical significance.

The in vitro transducing properties of AAV.Bov.Guelph and AAV.Po.Guelph were evaluated in a panel of mammalian cell lines including HeLa, HEK 293T, RLE, HT1080, PK-15, HEK293, BHK-21, Vero, A549, Dulac, MDBK, AML-12, MDBK, and polarized bovine epithelial cells. The human AAV serotype 6 was included as a benchmark for in vitro transduction performance, since AAV6 and AAV1 are considered among the best serotypes for the transduction of a wide variety of cell types [34]. The panel of cell lines was highly varied and consisted of several different species and cell types, including human-derived cell lines HeLa (cervical cancer), HEK 293T (kidney), HT1080 (fibrosarcoma), HEK293 (kidney), and A549 (lung adenocarcinoma); porcine cell lines PK-15 and Dulac (both kidney); a bovine kidney cell line (MDBK); and bovine polarized tracheal epithelial cells. In addition, Vero cells (African green monkey kidney), MDCK (canine kidney), and rodent BHK-21 (Syrian golden hamster kidney) and AML-12 (mouse liver) cells were also included in the panel. Interestingly, when infected at the same multiplicity of infection (MOI), AAV.Bov.Guelph demonstrated superior transduction in the majority of cell lines tested compared to both AAV6 and AAV.Po.Guelph. AAV.Bov.Guelph showed relative luciferase expression of at least an order of magnitude higher than AAV6 in many of the cell lines tested, including HeLa, 293T, RLE, HT1080, HEK293, Vero, Dulac, and polarized bovine trachea cells (Figure 4). In other cases, AAV.Bov.Guelph exhibited greater expression than AAV6 but with less than a 10-fold increase. This was evident for PK-15, BHK-21, A549, and AML-12 cell lines, with AAV.Bov.Guelph showing a modest improvement over AAV6 in these lines. Interestingly, luciferase expression in MDBK bovine cells was lower than that in human cell lines 293T, HeLa, and HT1080 for AAV.Bov.Guelph. However, this was still at least an order of magnitude higher than AAV6 luciferase expression in this cell line. In addition, AAV.Bov.Guelph transduced polarized bovine trachea cells, while neither AAV6 nor AAV.Po.Guelph was able to transduce these cells to any degree. 

Overall, AAV.Po.Guelph was a poor in vitro transducer, even in porcine cell lines PK-15 and Dulac cells. Notable expression for AAV.Po.Guelph was only observed for HeLa, HEK 293T, HT1080, PK-15, and HEK 293 cells. Interestingly, most of these cells were human derived, whereas no transduction was observed for any non-human cell line except for the porcine PK-15 cells.

### 3.5. Analysis of AAV.Po.Guelph and AAV.Bov.Guelph Expressing Luciferase in Mice following Intranasal Administration

**Figure 5:**
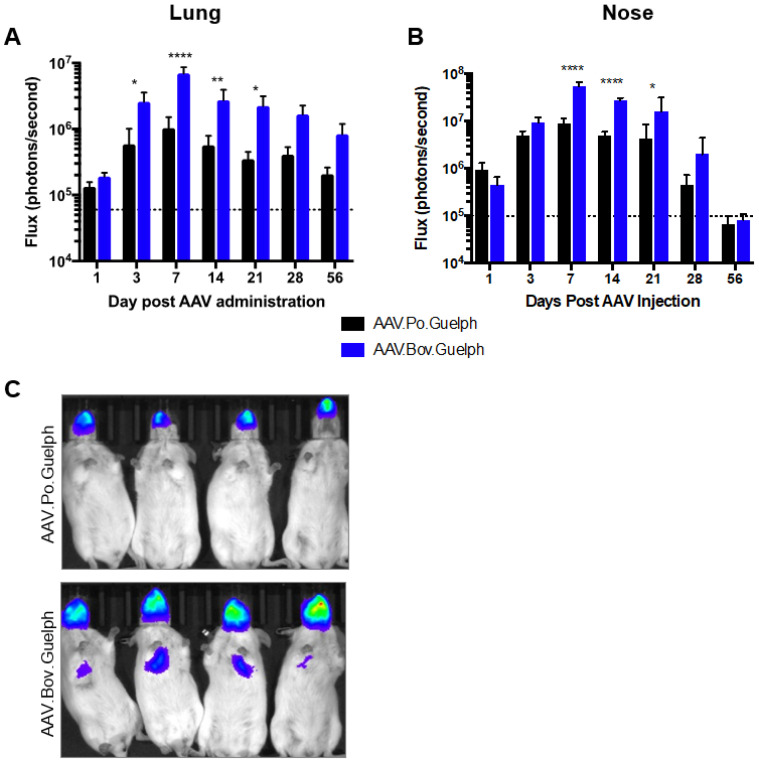
AAV.Po.Guelph and AAV.Bov.Guelph transduction of the mouse airway. A total of 1 × 10^11^ vg of AAV.Po.Guelph-Luciferase or AAV.Bov.Guelph-Luciferase was administered intranasally to six-week-old female Balb/c mice (n = 4/group). Luciferase expression in the (**A**) lungs and (**B**) nose was quantified on days 1, 3, 7, 14, 21, 28, and 56 after AAV administration. The dashed lines indicate the background threshold. **** *p* < 0.0001, ** *p* < 0,01, and * *p* < 0.05. (**C**) Images from three days after intranasal administration of AAV.

AAV.Po.Guelph and AAV.Bov.Guelph expressing firefly luciferase were administered to mice intranasally at a dose of 1 × 10^11^ vg. Luciferase expression in the lungs and the nose was quantified on days 1, 3, 7, 14, 21, 28, and 56 after AAV administration (Figure 5). In the lungs, both vectors resulted in peak luciferase expression on day 7 post-administration. However, AAV.Bov.Guelph significantly outperformed AAV.Po.Guelph on days 3 to 21 post-administration, with its peak luciferase expression being almost 10-fold higher than that of AAV.Po.Guelph (Figure 5A). In the nose, a similar pattern was observed, with peak luciferase expression occurring on day 7 post-administration for both vectors. As in the lungs, AAV.Bov.Guelph resulted in significantly higher luciferase expression in the nose as compared to AAV.Po.Guelph from day 7 to 21 post-administration (Figure 5B). An in vivo luciferase imaging system was used to visualize luciferase expression in the mice and confirmed this trend (Figure 5C).

### 3.6. Analysis of AAV.Po.Guelph and AAV.Bov.Guelph Expressing Luciferase in Mice following Intraperitoneal Administration

**Figure 6:**
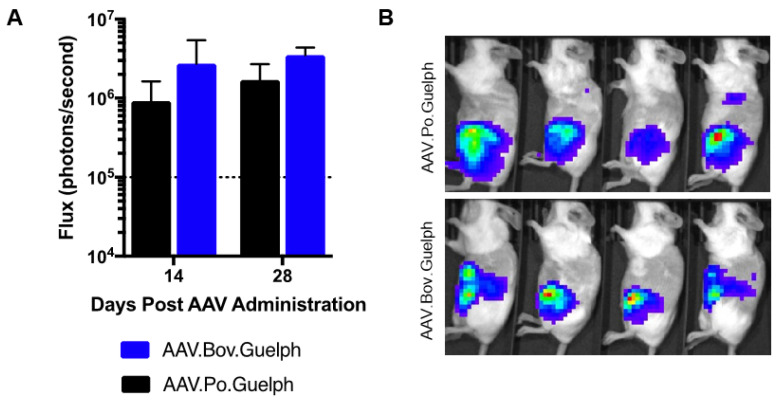
AAV.Po.Guelph and AAV.Bov.Guelph in vivo transduction profile following intraperitoneal administration. A total of 1 × 10^11^ vg of AAV.Po.Guelph-Luciferase or AAV.Bov.Guelph-Luciferase was injected IP to BALB/c mice (n = 4/ group). (**A**) The luciferase signal (left lateral) was quantified on days 14 and 28 after AAV administration. The dashed lines indicate the background threshold. (**B**) Images demonstrating distribution of luciferase expression on day 28 after IP injection.

To evaluate the transducing properties of AAV.Po.Guelph and AAV.Bov.Guelph expressing firefly luciferase when administered systemically, groups of mice (n = 4) were intraperitoneally injected with 1 × 10^11^ vg of vector, and luciferase expression in the peritoneal cavity was quantified on days 14 and 28 post-administration (Figure 6A), with the in vivo image obtained on day 28 shown (Figure 6B). No significant differences were observed between vectors at either timepoint. Confirmation of the liver as the target organ for these capsids was confirmed histologically (Figure S4).

## 4. Discussion

Here, we describe the isolation of two novel AAV capsids from non-human species and characterize their in vitro and in vivo transducing properties. The porcine AAV capsid, designated AAV.Po.Guelph, appears to be the product of multiple recombination events between both porcine and human AAVs, whereas AAV.Bov.Guelph was isolated as a contaminant of a caprine adenovirus stock, demonstrating the potential for bovine AAVs to infect caprine species.

As demonstrated by the numerous recombination events described herein, the AAV capsid may readily undergo recombination. It has previously been shown that defects in the AAV capsid may be complemented by a co-infecting AAV, as was the case when Bowles and colleagues were able to rescue mutant AAV2 capsids with AAV3 [35]. Recombination may be particularly advantageous in the case of the cap gene, where the exchange of genetic material with other AAV species may yield novel capsid characteristics. Such characteristics might include the ability to bind to heparin sulfate proteoglycan; the switching of epitopes from one recognized by pre-existing antibodies to one that the host might be naive to; changes in the ability to bind receptors or co-receptors, thereby resulting in novel cell or tissue tropisms; and potentially other useful properties. A previous study investigating the diversity of primate-derived AAVs has shown that even minor changes in the capsid might lead to divergent serological reactivity or transduction efficiency [36]. Furthermore, the authors concluded that homologous recombination is a powerful force shaping the evolution and sequence diversity of AAV capsid sequences. The apparently frequent recombination events between human and porcine AAV capsids suggest recurrent co-infection of porcine and human serotypes in the same cell. This likely occurs in pigs more frequently than it does in humans since pre-existing immunity to AAVpo1 did not appear to occur in pooled human sera [30], whereas porcine sera robustly prevented transduction by all human AAV serotypes tested [37]. This suggests that the pig may be acting as a sort of mixing vessel for various human and non-human AAV species and may represent a treasure trove for the isolation of novel AAV capsid sequences. 

In the case of AAV.Bov.Guelph, two amino acid changes differentiated it from bovine AAV: I124L and R499G. The change from isoleucine to leucine conserves the non-polar properties of isoleucine and thus would not be expected to have a substantial effect on capsid protein folding. On the other hand, the change from the positively charged amino acid arginine to glycine, an uncharged and relatively small amino acid, may influence the local protein secondary structure. In addition, the arginine-to-glycine change occurs in a variable region known to play a role in virus neutralization, heparin binding, and cell transduction [33]. 

In vitro transduction assays using a panel of mammalian cells demonstrated AAV.Bov.Guelph’s superior transduction properties in many commonly used cell lines including HeLa, HEK293T, HEK293, and Vero. This was expected, as Schmidt et al. found that the closely related bovine AAV was a strong transducer of HEK293T and HeLa cells [13]. In addition, AAV.Bov.Guelph demonstrated excellent transduction of respiratory tract-derived cell lines RNLE, A549, and polarized bovine tracheal epithelial cells. The diversity of transducible cell lines can be explained by their expected receptors. Schmidt and Chiorini previously identified gangliosides as bovine AAV’s most probable receptor [38]. Given how closely related AAV.Bov.Guelph is to bovine AAV, it is likely that gangliosides are also AAV.Bov.Guelph’s receptor. Since gangliosides are ubiquitously expressed in all mammalian cells, it is unsurprising that AAV.Bov.Guelph was able transduce the panel of mammalian cells [39]. 

In vivo, AAV.Bov.Guelph delivered the luciferase gene efficiently to the lungs and noses of mice after intranasal administration. Additionally, AAV.Bov.Guelph mediated luciferase expression following intraperitoneal injection. Interestingly, when Pasquale et al. used bovine AAV administered intranasally to deliver luciferase to mice, only modest luciferase activity was observed [40]. They were able to significantly increase luciferase expression by administering tannic acid prior to bovine AAV, which they attributed to tannic acid’s ability to redirect AAV particles entering the transcytosis pathway to the transduction pathway [41]. Given that AAV.Bov.Guelph was able to achieve moderate luciferase expression without tannic acid, it is possible that the slight changes in capsid amino acids could impact its ability to undergo the transduction pathway as opposed to the transcytosis pathway; however, a head-to-head comparison of the two bovine capsids is needed in order to draw any conclusions. Overall, AAV.Bov.Guelph may be a useful capsid for respiratory tract directed gene therapy. 

AAV.Po.Guelph possesses a four-amino-acid insertion at position 529 relative to AAVpo4, starting with two amino acids with positively charged side chains, followed by a polar amino acid, and ending with a non-polar amino acid. It is unclear what the significance of this insertion might be, but the insertion occurs in a region implicated in cell transduction, neutralization by pooled human sera, and neutralization by intravenous immunoglobulin. 

In vitro, AAV.Po.Guelph performed relatively poorly, with the highest transduction observed in the human-derived cell lines HeLa and HEK293T and low to no transduction in the rest of the cell lines tested. Its closest relative capsid-wise, AAVpo4, possessed a similar transduction profile when tested on a panel of mammalian cells. Like AAV.Po.Guelph, AAVpo4 did not significantly transduce MDCK, Vero, HEK293, or A549 cells but showed strong transduction in the porcine retina cell line, VR1BL [42].

In vivo, AAV.Po.Guelph was able to transduce the lung, nose, and peritoneal cavity of mice after intranasal or intraperitoneal administration. Higher luciferase expression was observed in the nose than in the lung. Interestingly, AAVpo4 also showed poor lung transduction after intranasal administration [31]. AAV.Po.Guelph-mediated luciferase expression was observed in the peritoneal cavity and was not significantly different from AAV.Bov.Guelph-mediated expression. Bello et al. found that AAVpo4 can transduce the liver efficiently [31], and histological images from AAV.Po.Guelph-administered mice showed evidence of liver transduction. Indeed, most AAV serotypes show liver transduction when administered intravenously and intraperitoneally [43,44]. 

## 5. Conclusions

Overall, two novel capsids, AAV.Bov.Guelph and AAV.Po.Guelph, were identified. Phylogenetic analyses were performed, and transduction profiles were established both in vitro and in vivo. Future biodistribution studies should be performed to elucidate specific capsid tropism, and these capsids should be compared to commonly used rAAV capsids to evaluate their potential as novel gene therapy vectors.

## Figures and Tables

**Table 1:** Primers and PCR conditions used for screening samples for the presence of AAV sequences.

	Round 1—Initial PCR	Round 2—Hemi-Nested PCR
Forward Primer	5′-ATGNTNATNTGGTGGGA GGA-3′	5′-ACCTTNGAACACCAGCAGC-3′
Reverse Primer	5′-CCANNNGGAATCGCAATGCCAAT-3′	
PCR Components	20 µL Platinum PCR Supermix	20 µL Platinum PCR Supermix
	1 µL (2 µM) Primers (Each)	1 µL (2 µM) Primers (Each)
	200 ng DNA	2 uL of PCR Product
PCR Conditions	94 °C—2 min	94 °C—2 min
	94 °C—30 s × 40 cycles	94 °C—30 s × 30 cycles
	55 °C—30 s × 40 cycles	55 °C—30 s × 30 cycles
	72 °C—30 s × 40 cycles	72 °C—30 s × 30 cycles
	72 °C—2 min	72 °C—2 min

**Table 2:** Primers and PCR conditions used for amplifying full-length capsid genes for porcine and bovine AAVs.

	Round 1—Initial PCR	Round 2—Hemi-Nested PCR
Porcine Capsid Forward Primer	5′-ATGTCGTTTGTTGATCACC-3′	5′-TTACAGGTTGCGGGTGAAGGTAGC-3′
Porcine Capsid Reverse Primer	5′-TTACAGRTTRCGRGTRAGGTAGC-3′	
PCR Components	20 µL Platinum PCR Supermix	20 µL Platinum PCR Supermix
	1 µL (2 µM) Primers (Each)	1 µL (2 µM) Primers (Each)
	200 ng DNA	4 µL of PCR Product
PCR Conditions	94 °C—2 min	
	94 °C—15 s	
	70 °C (−0.3 °C per cycle) 30 s	34 cycles
	72 °C—2.5 min	
	94 °C—15 s	
	55 °C—30 s	14 cycles
	72 °C—2.5 min	
	72 °C—5 min	

**Table 3:** An overview of tissue samples collected for AAV sequence isolation.

Type of Sample	Canine	Feline	Bovine	Porcine	Ovine	Equine	Exotic	Total
Extraction Method	Qiagen	Qiagen	Qiagen	MagNA Pure	Qiagen	Qiagen	Qiagen	–
Number Screened	10	4	8	74	2	3	3	104
Positive PCR Screening	1	0	0	46	2	1	1	51

**Table 4:** Changes in AAV.Po.Guelph relative to the next most similar AAV capsid, AAVpo4.

Change	Position	Variable Region ^1^	Role in AAV2 Capsid (Govindasamy et al., 2006) ^2^
A to P	182	None	NA
G to S	207	None	NA
D to E	327	II (262–268)	Transduction
Q to H	385	III (326–330)	Transduction and A20 neutralization
A to Q	449	IV (449–468)	HS and IVIG neutralization
G to N	450	IV (449–468)	HS and IVIG neutralization
A to G	453	IV (449–468)	HS and IVIG neutralization
T to S	461	IV (449–468)	HS and IVIG neutralization
T to A	498	V (487–504)	Transduction, HB, HS, and IVIG neutralization
R to K	500	V (487–504)	Transduction, HB, HS, and IVIG neutralization
D to N	507	None	NA
RKTV	529	VI (525–541)	Transduction, HS, and IVIG neutralization
A to T	544	VII (544–556)	A20, HS, and IVIG neutralization
S to G	582	VIII (579–594)	HB, HS, and IVIG neutralization

^1^ AAV2 VP1 numbering. ^2^ A20, AAV2 capsid antibody; IVIG, intravenous immunoglobulin G; HB, heparin binding; HS, pooled human sera. Variable region role based on description in Govindasamy et al., 2006 [33].

## Data Availability

All data generated or analyzed during this study can be found within the published article and its Supplementary Files. Sequences for AAV.Bov.Guelph (OR759014) and AAV.Po.Guelph OR759015) have been deposited in Genbank.

## Supplementary Materials

The following supporting information can be downloaded at: https://www.mdpi.com/article/10.3390/v16010057/s1, Figure S1: Distribution of AAV.Po.Guelph and AAV.Bov.Guelph vectors during production; Figure S2: Predicted phylogenic relationships between AAV.Po.Guelph capsid and other AAV capsid isolates; Figure S3: Predicted phylogenic relationships between AAV.Po.Guelph capsid and other AAV capsid isolates, continued; Figure S4: Alkaline phosphatase expression in liver tissue mediated by AAV.Po.Guelph and AAV.Bov.Guelph vectors following intravenous injection.
