# Supplementary material for: AAV Vectors Pseudotyped with Capsids from Porcine and Bovine Species Mediate In Vitro and In Vivo Gene Delivery
Source: Viruses. 2023 Dec 29;16(1):57. doi: 10.3390/v16010057 (PMC10820940; doi:10.3390/v16010057)
Supplement: Supplementary file 1 [file viruses-16-00057-s001.zip › viruses-2730222-supplementary.pdf]

Supplementary Figures

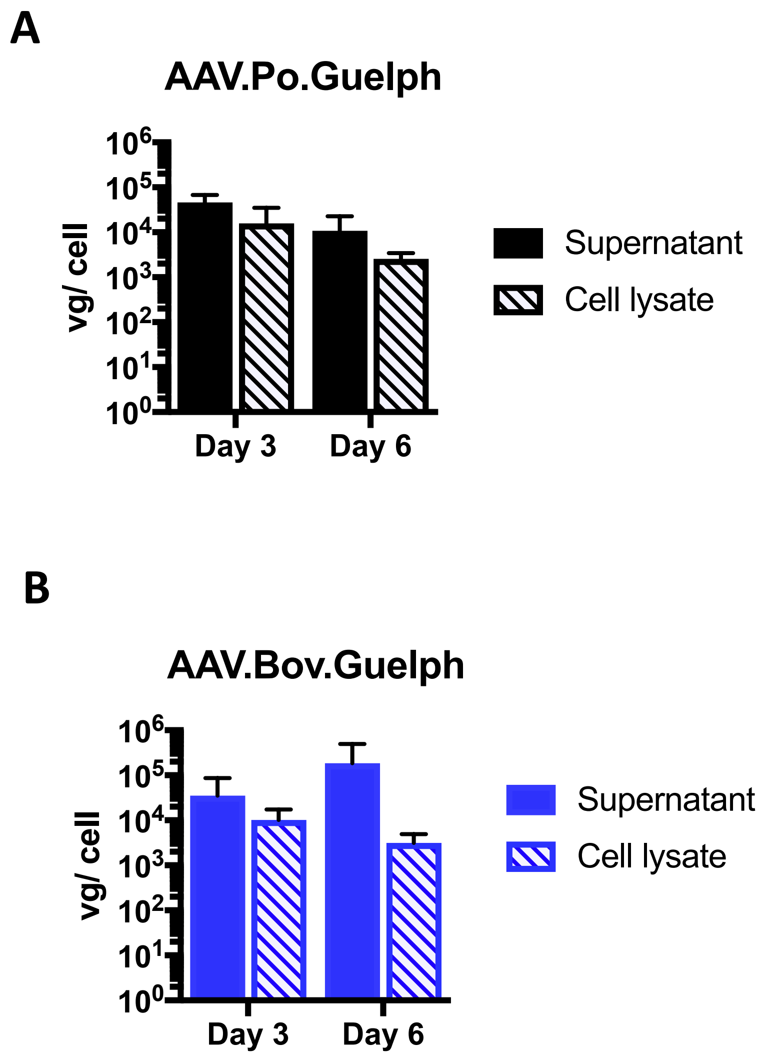

Figure S1. Distribution of AAV.Po.Guelph and AAV.Bov.Guelph vectors during production. Supernatant and cell lysate samples were collected on day 3 and 6 post triple transfection for vector production. AAV DNA extraction and vector genome quantification by qPCR to determine the proportion of vector produced in each fraction. There were no significant differences in vector accumulation between the cell lysate and supernatant.

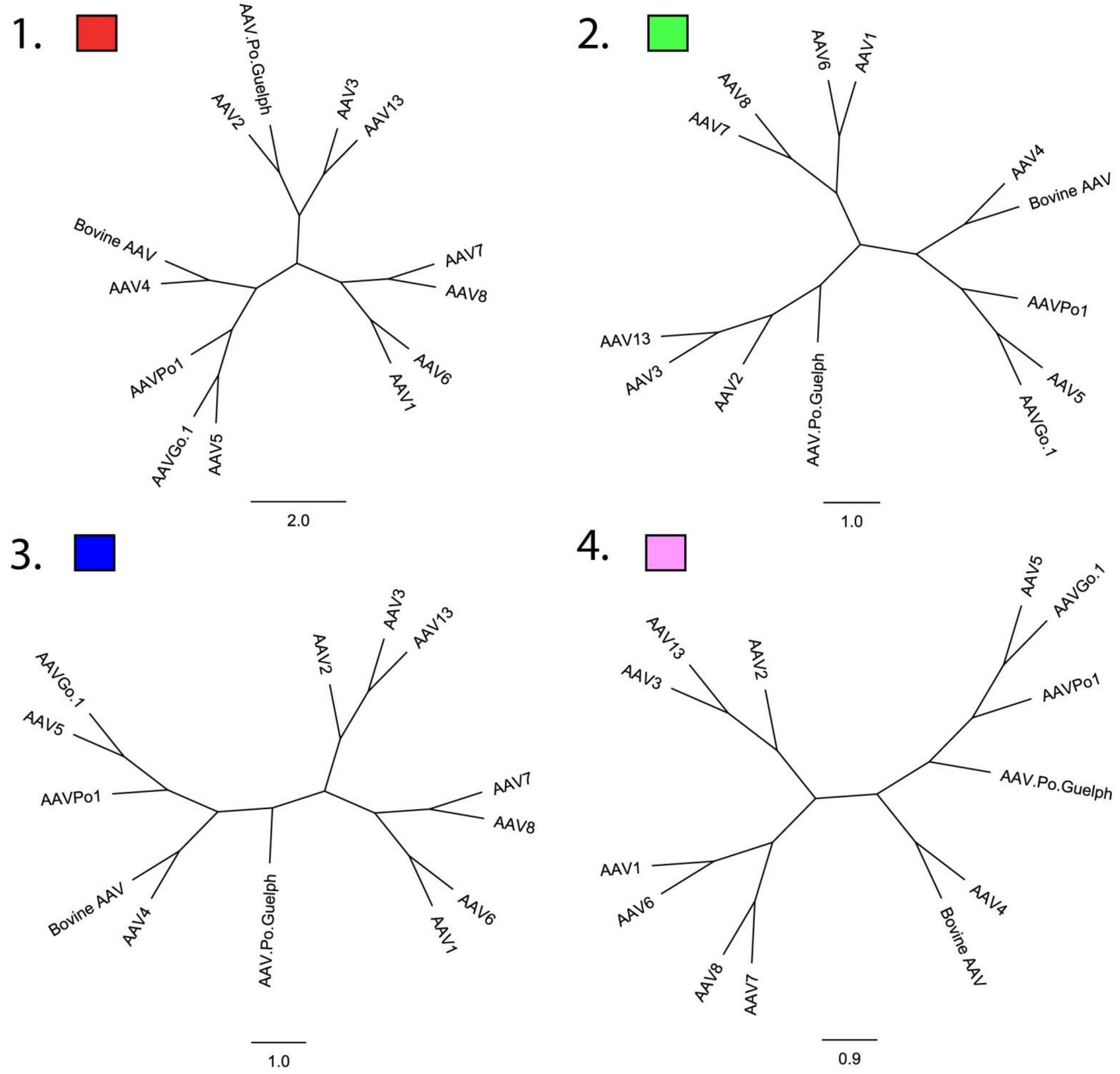

Figure S2. Predicted phylogenic relationships between AAV.Po.Guelph capsid and other AAV capsid isolates. Tree topologies 1 through 4. The probabilities of the various topologies are shown in figure 5.4. Topology 1 (red) is most likely for regions C, F, I, topology 2 (green) is most likely for regions E and K, topology 3 (blue) is most likely for regions D and G, and topology 4 (pink) is most likely for region B.

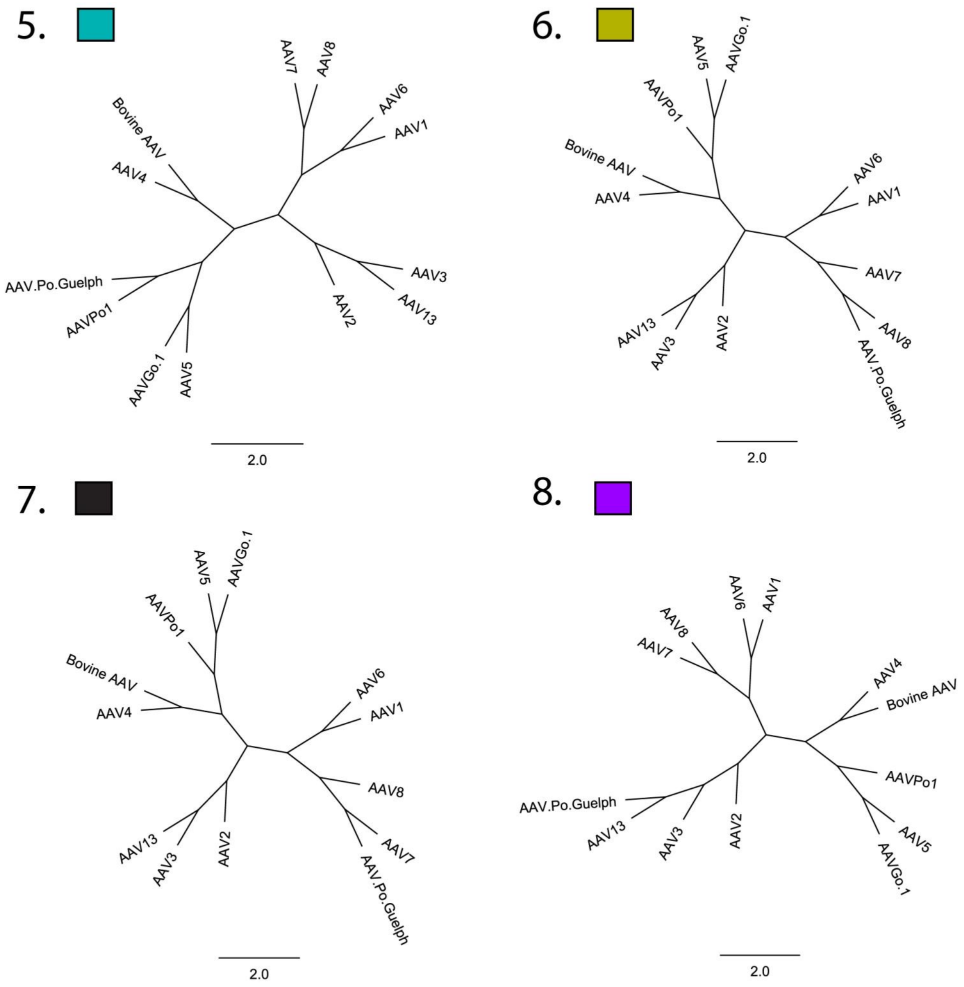

Figure S3. Predicted phylogenic relationships between AAV.Po.Guelph capsid and other AAV capsid isolates, continued: tree topologies 5 through 8. The probabilities of the various topologies for particular segments of the capsid gene are shown in figure 5.4. Topology 5 (turquoise) is most likely for region A, topology 6 (gold) is most likely for region J, topology 7 (black) is most likely for region H. Topology 8 was only likely for a small area within region C and was less probable than topology 1, and therefore a recombination event at this section does not seem likely.

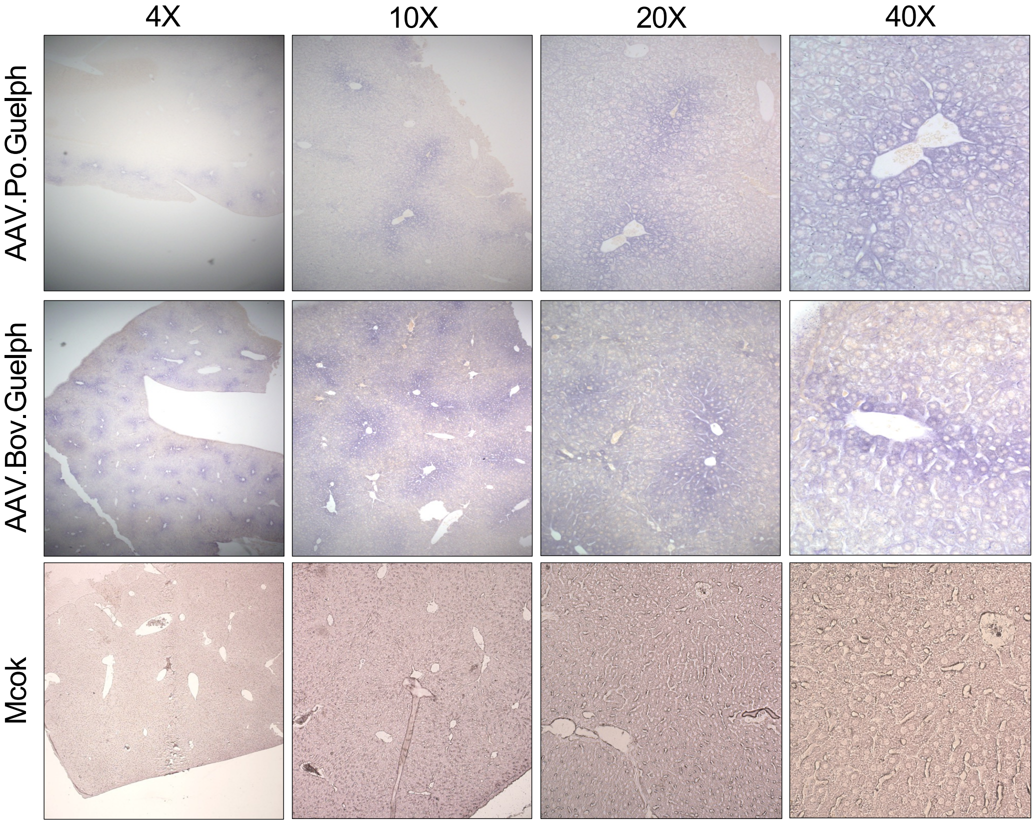

Figure S4. Alkaline phosphatase expression in liver tissue mediated by AAV.Po.Guelph and AAV.Bov.Guelph vectors following intravenous injection. $7x10^{10}$vg of AAV.Po.Guelph-AP, $2x10^{10}$vg of AAV.Bov.Guelph-AP or PBS was administered systemically to Balb/c mice (n=4) by tail vein injections. After 21 days, livers were harvested and processed for AP staining.
